# Glossopharyngeal neuralgia secondary to vascular compression in a patient with multiple sclerosis: a case report

**DOI:** 10.1186/1752-1947-6-213

**Published:** 2012-07-19

**Authors:** Emil Gaitour, Saeed Talebzadeh Nick, Charles Roberts, Eduardo Gonzalez-Toledo, Sai Munjampalli, Alireza Minagar, Bruce Vrooman, Dmitri Souzdalnitski, Behrouz Zamnifekri

**Affiliations:** 1 Pain Management Department, Cleveland Clinic, Cleveland, OH 44195, USA; 2 Department of Neurology, LSU Health Sciences Center, 1501 Kings Highway, Shreveport, LA 71130, USA; 3 Department of Anesthesiology, LSU Health Sciences Center, 1501 Kings Highway, Shreveport, LA 71130, USA; 4 Department of Radiology, LSU Health Sciences Center, 1501 Kings Highway, Shreveport, LA 71130, USA

## Abstract

**Introduction:**

Glossopharyngeal neuralgia is an uncommon, painful syndrome, characterized by paroxysms of pain in the sensory distribution of the 9th cranial nerve. Idiopathic glossopharyngeal neuralgia may be due to compression of the glossopharyngeal nerve by adjacent vessels, while secondary glossopharyngeal neuralgia is associated with identifiable lesions affecting the glossopharyngeal nerve at different levels of its neuroanatomic pathway. Glossopharyngeal neuralgia is rare in the general population, but is more common in patients with multiple sclerosis.

**Case presentation:**

A 56-year-old Caucasian woman with multiple sclerosis and migraine presented to our facility with intermittent lancinating pain to the right of her throat, tongue, and the floor of her mouth that had been occurring for the past year. The pain was intense, sharp, and stabbing, which lasted two to six seconds with radiation to the right ear. Initially, the attacks were infrequent, however, they had become more intense and frequent over time. Our patient reported weight loss, headache, painful swallowing, and the inability to maintain sleep due to painful attacks. A neurological examination revealed a right-handed woman with trigger points in the back of the tongue and throat on the right side. She also had dysphagia, hoarseness, and pain in the distribution of the right glossopharyngeal nerve. Mild right hemiparesis, hyperreflexia, dysmetria, and an ataxic gait were present. A magnetic resonance imaging scan of the brain was consistent with multiple sclerosis and magnetic resonance angiography demonstrated a loop of the posterior inferior cerebellar artery compressing the right glossopharyngeal nerve. She responded satisfactorily to carbamazepine. Microvascular decompression and Gamma Knife® radiosurgery were discussed in case of failure of the medical treatment; however, she declined these options.

**Conclusions:**

Glossopharyngeal neuralgia in multiple sclerosis may occur due to vascular compressive lesions and it should not be solely attributed to the underlying demyelinating process. Vascular compression of the glossopharyngeal nerve could independently cause glossopharyngeal neuralgia in patients with multiple sclerosis, and vascular imaging to exclude such a diagnosis is recommended.

## Introduction

Glossopharyngeal neuralgia (GPN) is a rare painful syndrome, which is characterized by paroxysms of pain in the sensory distribution of the 9th cranial nerve. Idiopathic (essential) GPN is caused by compression of the glossopharyngeal nerve by adjacent vessels; secondary (symptomatic) GPN is associated with various lesions that affect the glossopharyngeal nerve at different levels of its path, such as brainstem structures, cisternal branches, jugular foramen, as well as its extracranial divisions. The incidence of GPN is about 0.7 per 100,000 people in the general population [1]. Like trigeminal neuralgia (TN), which occurs more often in patients with multiple sclerosis (MS), GPN is also more common in the MS population than in the general population. However, only 0.5 per 1000 patients with MS develop GPN [1]. Here, we present the case of a patient with MS and GPN who also had vascular compression of the glossopharyngeal nerve by the posterior inferior cerebellar artery.

## Case presentation

A 56-year-old right-handed Caucasian woman with long-standing MS presented to our facility with intense, intermittent pain in the right side of her tongue and floor of her mouth, which was precipitated by speaking, chewing, and swallowing. The episodic pain was intense, sharp, and with a lancinating character, and lasted from two to six seconds. She developed the pain one year prior to her last visit and later on she also experienced vocal hoarseness. The pain was localized to the posterior part of her tongue and surrounding areas on her right side. On certain occasions the pain radiated to her right ear. The laryngeal pain had an episodic nature and between attacks our patient was free from pain. Initially she had a few attacks of this pain per week, however, the frequency and intensity of the pain gradually increased. At the end of the year she noticed that her voice became significantly hoarse and the attacks were triggered not only by swallowing but also by speaking. She modified and restricted her diet to only soft food and liquids, with only temporary relief. During the last two weeks prior to her visit the throat pain recurred regularly with eating and speaking. She saw her primary care physician, who prescribed hydrocodone with only partial relief.

She was diagnosed with MS and migraine 18 years prior to case presentation. At the time of her new head and neck pain she was on interferon-β1a 30 μg intramuscularly once weekly.

A neurological examination revealed a middle-aged woman with stable vital signs and normal mental status. She was oriented to all cognitive spheres with no aphasia, apraxia, or agnosia. During the assessment she was in pain off and on, and pain was precipitated by speaking. A cranial nerve examination revealed dysphagia, vocal hoarseness, and pain in the distribution of the right glossopharyngeal nerve (posterior third of the tongue, floor of the mouth, and right side of the throat). Her palate was symmetrically moving upon phonation and her gag reflex was present. Our patient had difficulty with differentiating basic tastes, particularly salty tastes on the right side of the posterior of her tongue during the painful attacks. Normal sensation in the distribution of cranial nerve IX was present. She was also right hemiparetic with a wide-based stance and ataxic gait. Her deep tendon reflexes were depressed and her plantar responses were flexor.

A speech pathologist evaluated our patient and could not determine any sign of aspiration; however, a soft mechanical diet with chopped meat, feeding at 90 degrees, with small boluses and maintained in the sitting position with head elevated to avoid and aspiration for 30 minutes.

**Figure 1:**
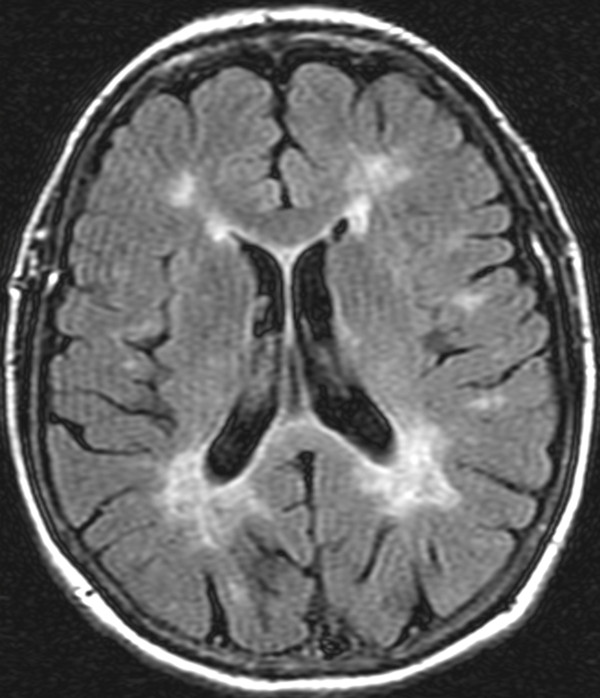
**Magnetic resonance imaging scans: fluid attenuated inversion recovery (FLAIR) sequence, transverse plane.** Shown are demyelinating areas involving the periventricular areas of the lateral ventricles, more prominent in the posterior. A similar demyelination process is present in the white matter of the brain sulci (medullary white matter).

**Figure 2:**
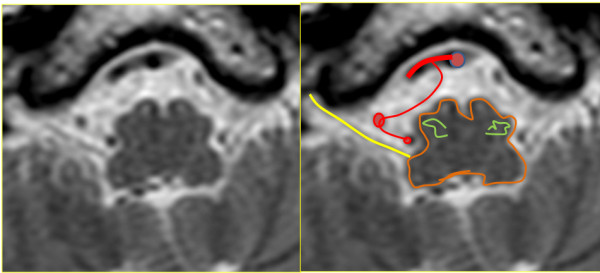
**Magnetic resonance imaging scans: high-resolution, T2-weighted, transverse plane.** Vascular contact between the glossopharyngeal nerve (yellow) exiting the retro-olivary fossa (olive green) and right posterior inferior cerebellar artery (PICA).

**Figure 3:**
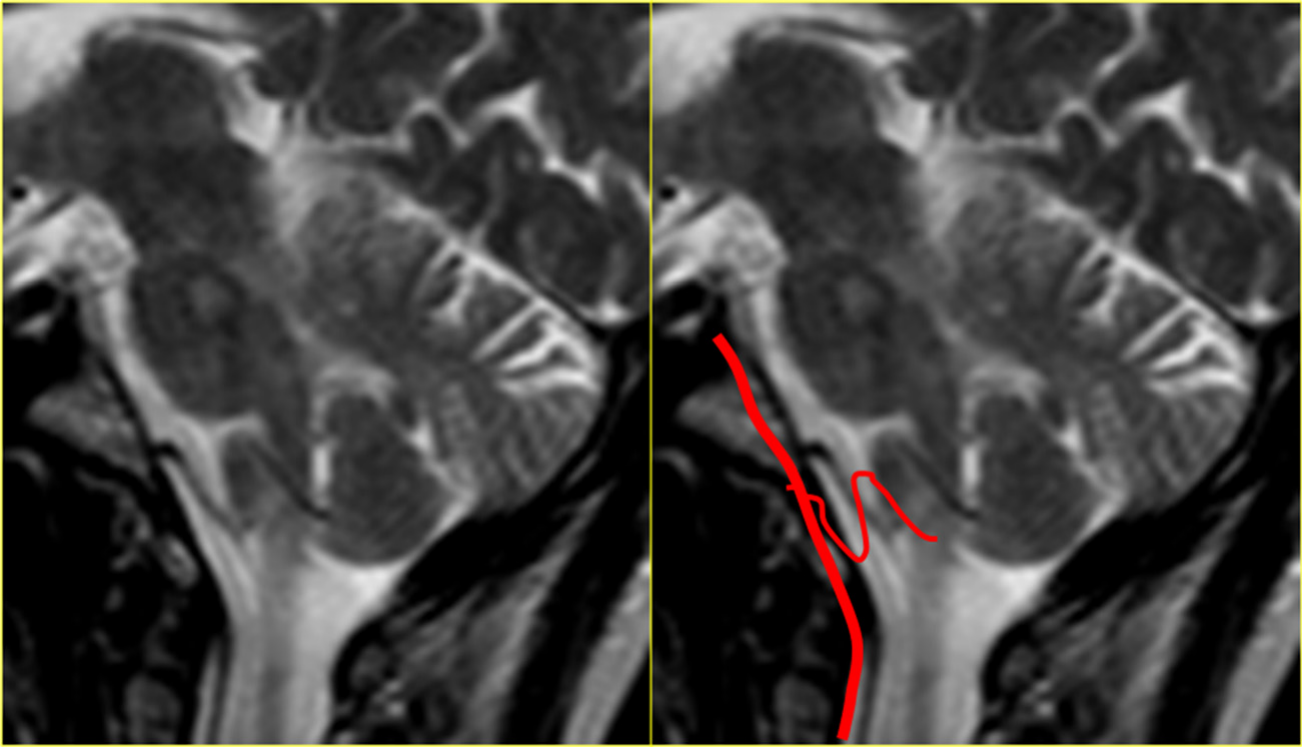
**Magnetic resonance imaging scans: T2-weighted image, sagittal plane.** Scan shows the vertebrobasilar system. The posterior inferior cerebellar artery (PICA) arises from the vertebral artery. The upward buckle almost reaches the caudal pons.

**Figure 4:**
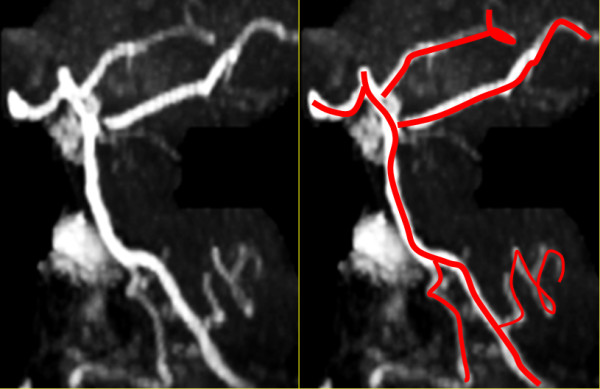
**Magnetic resonance angiogram.** The posterior inferior cerebellar artery (PICA) originates from the right vertebral artery. The left vertebral artery is underdeveloped.

A magnetic resonance imaging (MRI) scan of the brain with and without contrast and a magnetic resonance angiogram of the head and neck vessels were performed. On axial fluid attenuated inversion recovery (FLAIR) MRI scans of the brain, multiple hyperintense lesions were present in the periventricular areas (Figure 1), medulla, left side of pons, right middle cerebellar peduncle, the left cerebellar dentate nucleus, and in the prerubral area of the midbrain. On T1-weighted images a number of hypointense lesions consistent with T1-weighted black holes were present. No post-contrast T1-weighted enhancing lesions were present. Corpus callosum atrophy was absent. On high-resolution T2-weighted imaging, vascular contact between the glossopharyngeal nerve and the right posterior inferior cerebellar artery (PICA) was observed (Figures 2 and 3). A magnetic resonance angiogram of the head revealed the posterior inferior cerebellar artery originating from the right vertebral artery and that the left vertebral artery was underdeveloped, along with compression of the right glossopharyngeal (GP) nerve by the PICA (Figure 4).

A soft diet with hydration using intravenous 5% dextrose in half amount of normal saline 1000ml intravenously daily at a rate of 60ml/h was initiated. Treatment included methylprednisolone 1g intravenously daily for three days, carbamazepine 200mg orally twice a day, and paracetamol 650mg orally every six hours as needed.

At the end of the second day our patient reported a marked improvement in pain with absence of pain at nighttime and during sleep. Her speech improved and she requested to advance her diet from soft to mechanical soft.

While on carbamazepine, she reported reasonable control of her glossopharyngeal pain. She also reported that once she missed a dose of her medications, the pain rapidly recurred. The options for surgical intervention such as microvascular decompression versus Gamma Knife® treatment were discussed with our patient in case medical treatment failed; however, she rejected any further interventions. Our patient was discharged on carbamazepine.

## Discussion

GPN is a distinctive but rare painful syndrome. The incidence of GPN-like trigeminal neuralgia is higher among patients with MS than in the general population [2,3]. Trigeminal neuralgia occurs much more commonly in patients with MS (4000 per 100,000) [4] than in the general population (12.6 per 100,000) [3].

Usually the incidence of GPN increases with age and most often affects persons older than 50 years; however, in patients with MS, GPN tends to occur at a younger age [4]. Our patient had longstanding MS; however, she developed GPN at the age of 56, pointing towards potential secondary causes of GPN. The distribution of the pain in GPN can be different. It can be otitic (pain in and around the ear, mastoid region) or oropharyngeal (pain in the side or back wall of the pharynx, posterior third of the tongue, fauces, tonsil, or soft palate) similar to our patient. Otitic pain does not often radiate, while oropharyngeal pain radiates to the ear or adjacent regions in the majority of cases, as was the case with our patient. The pain is described as ‘burning’ and less frequently as ‘stabbing’,‘cutting’ or ‘like needles’. Occasionally it manifests as a ‘continuous ache’, a ‘pressure pain’, ‘throbbing’ or ‘sticking’. There can be considerable variation in the mode of onset and frequency and duration of attacks. Most often the onset is sudden and spontaneous, however, a gradual onset of an increasingly severe pain also occurs. The attacks tend to occur with a frequency of one to five per year. The attacks usually last from several weeks to one to two months with pain-free days during the attack period. The duration of the neuralgic pain is usually from seconds to minutes (up to 20 minutes). The number of these neuralgic pains in a 24-hour period commonly averages between five to thirty (up to one hundred) and they occur during day and night. The severity of the pain is also extremely variable (from mild to severe). Mild attacks occur and cause minor inconvenience. Usually patients are stable between the attacks, but some of them have mild, episodic ‘sticking sensations’ in the same area as the pain. The most commonly observed precipitating factor is swallowing (saliva, solid foods and liquids), which was present in our patient. Chewing and talking have also been described as triggers, as in our patient. Infrequent causes are yawning, movements of the head, straining and mouth movements. Certain foods, independent of the act of swallowing, may precipitate an attack (bitter, spicy, salty), but never sweet [5].

Other non-painful clinical manifestations include sensations of a swollen soft palate, headache, and hyperesthesia, which may occur for several hours, usually in a secondary field, after the pain has subsided. Other less frequent presentations of GPN consist of burning sensations in the muscles of the neck, difficulty in swallowing liquids and solids, excessive secretion of saliva, bilateral tinnitus, bilateral lacrimation, flushing and sweating, irritation in the throat and excessive coughing, dizzy attacks, objective vertigo and unsteadiness, bradycardia and even cardiac arrest with syncope [6].

The causes of GPN are diverse and are usually divided into two groups. Secondary underlying causes such as neoplasm or inflammatory diseases, such as MS, affect the glossopharyngeal nerve at different levels of its path at brainstem structures, cisternal branches, jugular foramen, and extracranial divisions. Development of GPN in MS is quite rare and most cases are attributed to the underlying demyelinating process. Minagar and Sheremata [4] reported a cohort of four patients with MS with GPN with no vascular compressive lesions of the glossopharyngeal nerve. Patients with GPN and without an identifiable underlying pathological process are referred to as having essential or idiopathic GPN. Idiopathic GPN according to some authors is caused by compression of cisternal part of the glossopharyngeal nerve by intracranial vessels [7]. For this reason microvascular decompression (MVD) is successful in the majority of the cases but not in all [8]. A three-dimensional MRI scan of the brain revealed that contact or contiguity between blood vessels and neural tissue is not visibly different on the symptomatic and asymptomatic sides, thus discounting any consistent causal relationship to pain [9]. In our patient, we demonstrated a definitive contact between the PICA and glossopharyngeal nerve that could explain our patient’s symptomatology. Diagnostic investigation for GPN should exclude MS and local compression, particularly by cancerous lesions, which require more aggressive treatments. Vascular compression is a common and potentially treatable cause of GPN but does not account for all previously designated idiopathic cases. The proposed mechanisms of irritation of the glossopharyngeal nerve and the resultant painful paroxysms include hyperexcitability of the glossopharyngeal nerve and ephaptic conduction; however, none have been fully explored. Based on current hypotheses, it is suggested that compression of the glossopharyngeal nerve at the nerve root entry zone results in demyelination and ephaptic transmission among the glossopharyngeal nerve axons; another theory suggests that vascular compression of a nerve induces repetitive activation of primary afferents in the nerve and leads to hyperactivity and hyperexcitability in the central neurons; the last implies activation of *N*-methyl- d-aspartic acid receptors in the compressed nerve [10]. In patients with MS demyelinating plaques at the root entry zone could be the potential cause of paroxysmal TN [11]. Most probably similar root entry zone lesions of the glossopharyngeal nerve are associated with ephaptic conduction, which may serve as an alternative explanation of GPN in our patient with MS.

Medical treatment of GPN with carbamazepine or gabapentin is effective in the majority of patients. Our patient responded well to carbamazepine at a moderate dose. In cases refractory to medical therapy surgical methods could be applied. Surgical methods include destruction of petrous ganglion (radiofrequency percutaneous rhizothomy, Gamma Knife® stereotactic radiosurgery), extracranial (neurotomy) or intracranial (rhizotomy) resection of glossopharyngeal nerve [12], separation of the vessel from the nerve (MVD) [13], destruction of glossopharyngeal nerve ascending fibers (tractotomy) [14], motor cortex stimulation, thalamic and other deep brain stimulation, and centrally administered opioids. More recently, endoscopy has been employed as the sole modality in glossopharyngeal nerve decompression [15].

## Conclusions

In summary, patients with MS who present with new-onset GPN must be thoroughly assessed for the presence of treatable causes of GPN and it should not be automatically attributed to the presence of demyelinating lesions that affect the glossopharyngeal nerve. We strongly recommend vascular imaging studies such as magnetic resonance angiography of the head and neck vessels in all patients with MS with GPN in order to diagnose or exclude vascular compressive lesions of the glossopharyngeal nerve.

## Consent

Written informed consent was obtained from the patient for publication of this manuscript and any accompanying images. A copy of the written consent is available for review by the Editor-in-Chief of this journal.

## Competing interests

The authors declare that they have no competing interests.

## Authors’ contributions

EG, AM, EGT, STN, and CR prepared the text and collected all the medical data. EG, BV and DS reviewed the literature, provided suitable references and assisted with the draft version of the paper. EGT, AM, and BZ reviewed and interpreted the MRI images and prepared them for the manuscript. SM and AM reviewed the paper and revised it to the final format. All authors read and approved the final manuscript.
